# The Impact of Deleting Stem-Loop 1 of Epstein–Barr Virus-Encoded RNA 1 on Cell Proliferation

**DOI:** 10.3390/v14112538

**Published:** 2022-11-16

**Authors:** Zubaida Hassan, Pretty S. Philip, Gulfaraz Khan

**Affiliations:** 1 Department of Medical Microbiology and Immunology, College of Medicine and Health Sciences, United Arab Emirates University, Al Ain P.O. Box 15551, United Arab Emirates; 2 Department of Microbiology, Faculty of Life Sciences, Modibbo Adama University, PMB 2076, Yola 640001, Nigeria

**Keywords:** CDT1, EBER1 structure, molecular mechanism, mutants, proliferation

## Abstract

Epstein–Barr virus-encoded RNAs (EBERs) are two small, noncoding, structurally conserved transcripts, constitutively expressed at >10^6^ copies per EBV-infected cell. They have been shown to drive cell growth. However, the mechanism(s) involved in EBER-induced proliferation is not clear. In this study, we investigated the molecular mechanisms and structural impact of EBER1. Sequences of EBER1 stem-loops (SL) 1, 3, and 4 were deleted, creating three mutants: ∆SL1, ∆SL3, and ∆SL4. These mutants were cloned into pHebo plasmids and expressed in Jurkat cell lines. Cells transfected with wildtype EBER1 and pHebo were used as controls. Cell proliferation was monitored by microscopy and flow cytometry. Microarray, qPCR, and Western blotting were used to investigate the cell cycle markers. We found significantly higher cell proliferation in wildtype EBER1 cells compared to pHebo, ∆SL1, and ∆SL3, but not ∆SL4 mutants. There was also significant upregulation of S-phase and G2/M phase markers in wildtype EBER1 and ∆SL4 mutant. Furthermore, CDT1, a factor for DNA replication, was upregulated in wildtype EBER1 and ∆SL4 mutant. However, in ∆SL1 mutant, CDT1 was significantly downregulated and translocated to the cytoplasm. These data indicate that the structure of EBER1 is important in cell proliferation.

## 1. Introduction

Epstein–Barr virus (EBV) is an oncogenic virus that infects and establishes latency in >90% of the human population worldwide. EBV infection is generally characterised into lytic and latent infections [1]. Most EBV-associated malignancies are primarily linked to latent infection [2]. During EBV latent infection, a limited number of viral genes are expressed. Based on the pattern of viral gene expression, EBV latent infection is subdivided into four programs referred to as Latency 0-III [2]. The genes that are common to all four programs are the noncoding RNAs, notably, EBV encoded RNAs (EBERs) [2,3,4]. EBERs are two small, structurally conserved noncoding transcripts that are expressed at >10^6^ copies per EBV-infected cell [5]. Noncoding RNAs are essential molecules in recoding cellular dynamics [6,7]. As such, EBERs could be potential oncogenes [8]. They are considered functional backups of the viral oncoprotein, latent membrane protein-1 (LMP-1) [9], and key molecules involved in EBV pathogenesis [10]. 

To understand the oncogenic potentials of EBERs, Yajima and colleagues [11] studied the impact of EBERs-deleted virus on cell growth and transformation in Akata cell lines. They found that, although the EBERs-deleted virus efficiently infected B lymphocytes, it had a significantly decreased growth rate and transforming ability compared to wildtype virus [11]. Furthermore, when they reconstituted EBERs at their native locus, the growth rate and transforming ability of the virus were restored [11]. This suggests, albeit indirectly, a possible role for EBERs in EBV-associated tumorigenesis. The direct impact of EBERs on enhancing growth potential was observed both in vitro in soft agar and in vivo in SCID mice [12]. Similarly, we have also demonstrated that EBER1 alone is capable of inducing cell growth in lymphoid cell lines [13]. 

Despite both direct and indirect evidence that EBERs could induce cell proliferation, which in turn could contribute to EBV-associated tumorigenesis [7,9,11,12,13,14,15], the molecular mechanism remains poorly understood. Some of the proposed mechanisms of EBERs-induced cell proliferation suggest rather indirect mechanisms, such as inhibiting the activity of protein kinase R (PKR) [12]. Likewise, pathways involving mitochondrial activity and intracellular Ca^2+^ have been suggested [13]. While most of the efforts to understand the mechanism of action of EBERs have focused on cellular dynamics, very few studies have examined the impact of the structure of EBERs on their function. It is well known that the biological function of an RNA largely depends on its structure [16,17]. It has also been observed that disrupting the structure of EBER2 abolishes more than 20% of its ability to induce gene expression [7]. Similarly, cells expressing mutated EBER1 have been found to have significantly reduced growth [14]. Therefore, the aim of this study was to investigate the structural impact and molecular mechanisms involved in EBER1-induced cell proliferation. We have identified CDT1 as a possible molecular marker that appeared to enhance EBER1-induced cell proliferation. Deleting SL1 resulted in eliminating the increased CDT1 expression and, thus, yielded comparable cell growth rates to the negative control cells.

## 2. Materials and Methods

### 2.1. Generation of EBER-1 Stem-Loop Mutants, Cloning and Transfection

**Figure 1:**
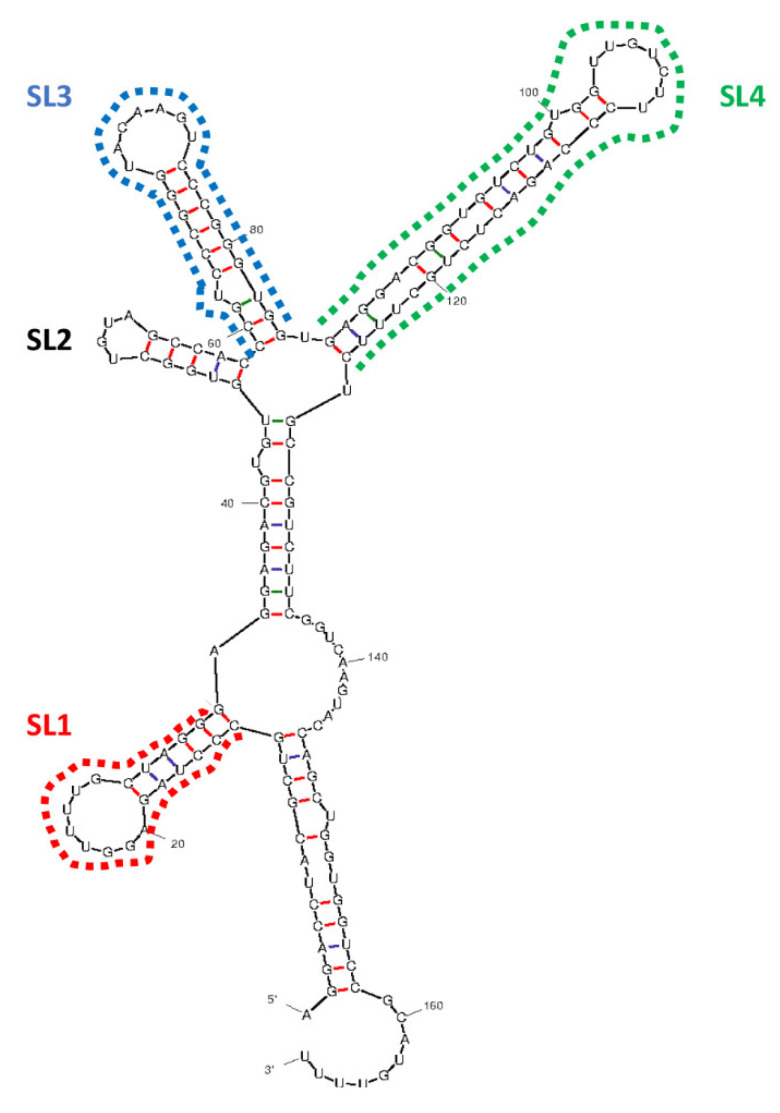
**Secondary structure of wildtype EBER1**. Secondary structure of EBER1 was determined using the mfold web server. Stem-loops that were deleted to create ∆SL1, ∆SL3, and ∆SL4 deletion mutants are indicated (dotted lines).

A spliced overlap extension PCR was used to generate stable stem-loop (SL) mutants by deleting, individually, three SLs (1,3,4) of EBER1 (Figure 1) [18]. EBER1 outer forward and reverse primers were flanked with *HindIII* and *BglII* restriction enzymes to control for insert orientation in pHebo plasmid during cloning. For subsequent PCRs, EBER1 outer primers (forward and reverse) were used for all mutants and the wildtype (Table S1). Cloning was performed using T4 DNA ligase (NEB, Hertfordshire, UK) according to the manufacturer’s instructions [3]. The clones were transfected into Jurkat cells by electroporation at 260 V and 950 µF (BioRad, Hercules, CA, USA) [19]. Hygromycin B was used as the selection marker. Transfection was confirmed by RT-PCR and in situ hybridisation [20]. Plasmid copy number was determined as previously described [21]. Briefly, using Avogadro’s formula, calibration curves were generated from qPCR data of a tenfold serial dilution of purified plasmid DNA for each construct (wildtype, ∆SL1, ∆SL3, and ∆SL4) [22]. Cellular expression of EBER1 and Hygromycin B genes from the transfectants were extrapolated from the calibration curves. The ratio of cDNA to its corresponding gDNA was calculated for each gene to account for the transcription efficiency. The mean expression of the two genes was used as the copy number.

### 2.2. Cell Culture

Jurkat cells were cultured in RPMI media supplemented with 1% L-Glutamine, 10% FBS, 0.1% Gentamycin, and 1% Penicillin-streptomycin (100 units/mL and 100 μg/mL, respectively). For stable cell lines of pHebo, EBER1, and mutants, 150 µg/mL of Hygromycin B (Gibco, Big Cabin, OK, USA) was added to the media. Cells were incubated at 37 °C in 5% CO_2_ until >90% confluence was reached. 

### 2.3. Monitoring Cell Growth and Viability

A total of 5 × 10^4^ viable cells were seeded in duplicates in a 12-well tissue culture plate containing 2 mL of media. The cells were grown under the same conditions as in cell culture. The cell growth rate was monitored every 24 h using trypan blue exclusion assay [23] for six days. pHebo transfected cell line was used as a negative control. At the end of the experiment (day 6), cells were collected for RNA and protein isolations. To ensure that the cell number determined using trypan blue exclusion dye was due to actual cell proliferation and not to differences in the rate of cell death, we stained the cells with 7AAD viability dye. Following a three- and six-day growth, cells were collected and washed twice in cold FACS buffer (10% FBS in PBS) at 5000× *g* for 5 min each. Pellets were resuspended in cold FACS buffer, a drop or two of 7AAD viability dye was added and mixed well, and absorbance was measured at 250 nm wavelength using flow cytometer (BD FACS Canto II, BD FACS Canto II, BD Bioscience, Franklin Lakes, NJ, USA).

### 2.4. Cell Cycle Analysis

Cell cycle analysis was performed as described previously [24]. Cells were collected at three- and six-days, washed twice in cold FACS buffer, and fixed in cold 80% ethanol for 15 min. The cells were washed with ice-cold FACS buffer and stained using either cycleTest™ plus DNA Reagent kit (BD Bioscience, Franklin Lakes, NJ, USA) according to the manufacturer’s instructions or a commercially available propidium iodide (PI) stain (BD Bioscience, USA). For PI protocol, cells were treated with RNase A at 37 °C for 10–15 min prior to PI staining. Ten thousand events were recorded per sample using a flow cytometer (BD FACS Canto II). Experiments were performed in duplicate and repeated four times. Cell cycle analysis was performed using the Watson (Pragmatic) model in FlowJo software v. 10.8.0 (BD Bioscience, USA). Genes involved in cell cycle progression/regulation were studied using qRT-PCR, and relative gene expression was determined using the 2^−ΔΔCT^ method [25]. qRT-PCR experiment was conducted in duplicate and repeated three times.

### 2.5. Subcellular Fractionation, Nucleic Acid Isolation, qRT-PCR, and Microarray

Subcellular fractions (nucleus and cytoplasm) were purified using the method of Greenberg and Bender [26] as previously described [27]. Genomic DNA was isolated using Qiagen DNA extraction kit (QIAamp^®^, Hilden, Germany). Total cellular and cytoplasmic RNA were isolated using TRIzol Reagent (Invitrogen, Waltham, MA, USA) protocols. A total of 5 µg RNA was DNase treated and reverse transcribed to cDNA using Promega A3500 kit (Promega, Madison, WI, USA) according to the manufacturer’s instructions. A total of 100 ng of gDNA/cDNA was amplified using power SYBR^®^ Green PCR master mix (Applied Biosystems, Waltham, MA, USA) in a 40-cycle reaction using QuantStudio^TM^ 7 Flex System (Applied Biosystems, Waltham, MA, USA). Melt curve analysis was performed. Spliced and unspliced β–actin was used to check for the quality of the fractionation. β–actin and spliced β–actin was used as loading controls and for the normalisation in qRT-PCR data analysis for total cellular and subcellular, respectively. qPCR-based microarray using a commercially available TaqMan^®^ 96-well DNA repair and human cell cycle and chromosomal replication microarray plates (Thermoscientific, Waltham, MA, USA) were performed according to the manufacturer’s instructions. Fold change was calculated relative to the wildtype EBER1, and at least 1-fold difference was considered significant for analysis. qRT-PCR experiments were performed in duplicates and repeated three times. Microarray experiments were performed according to the number of replicates available on the commercial plates and is indicated in the respective figure legends.

### 2.6. Isolation of Total and Subcellular Proteins and Western Blotting

Total cellular proteins were isolated by radioimmunoprecipitation assay (RIPA) buffer supplemented with 50 μL of β-mercaptoethanol and 1× protease inhibitor. Subcellular proteins were purified according to the protocol of Baghirova and colleagues [28]. The proteins from the cytosol and membrane-bound organelles were mixed in a 1:1 ratio and used as cytoplasmic proteins. Protein concentration was determined using the Bradford assay by measuring the absorbance at 595 nm wavelength. A total of 10–25 µg of proteins was separated on 10–12% SDS-polyacrylamide gel electrophoresis (PAGE) and transferred onto a nitrocellulose membrane [29]. The membrane was blocked in 5% BSA and immunoblotted with the desired antibody. A horseradish peroxide-conjugated secondary antibody specific to the primary antibody was then used for detection. Membranes were developed with ECL Western blotting substrate kit (Azure Biosystems, Dublin, CA, USA) according to the manufacturer’s instructions and imaged (Azure Biosystems, USA). Histone 3 and GAPDH were used as nuclear and cytoplasmic markers to check for the quality of the fractionation. GAPDH was used as the loading control and for normalisation in ImageJ data analysis for cytoplasmic protein expression. Each experiment was repeated three times.

### 2.7. Statistical Analysis

A haemocytometer was used to determine cell number, and trypan blue exclusion and 7AAD dyes were used for cell viability. Experiments were conducted in duplicates and repeated at least three times, unless otherwise stated. Data were expressed as means ± standard error of the mean (SEM) calculated using Microsoft Excel (Microsoft, Redmond, WA, USA). The difference between wildtype EBER1 and each of the mutants in each experiment was determined using a two-tailed student *t*-test assuming equal variance. *p*-values of less than or equal to 0.05 were considered statistically significant. Graphs were generated using Microsoft Excel (Microsoft, Redmond, WA, USA). A 2-way factorial ANOVA was used to compare the mean difference between the mutants and the wildtype for the six days using IBM SPSS statistics software 26.0 (Armonk, NY, USA).

## 3. Results

### 3.1. Prediction of RNA Structure, Confirmation of Deletion and Transfection, and Determination of Plasmid Copy Number

**Figure 2:**
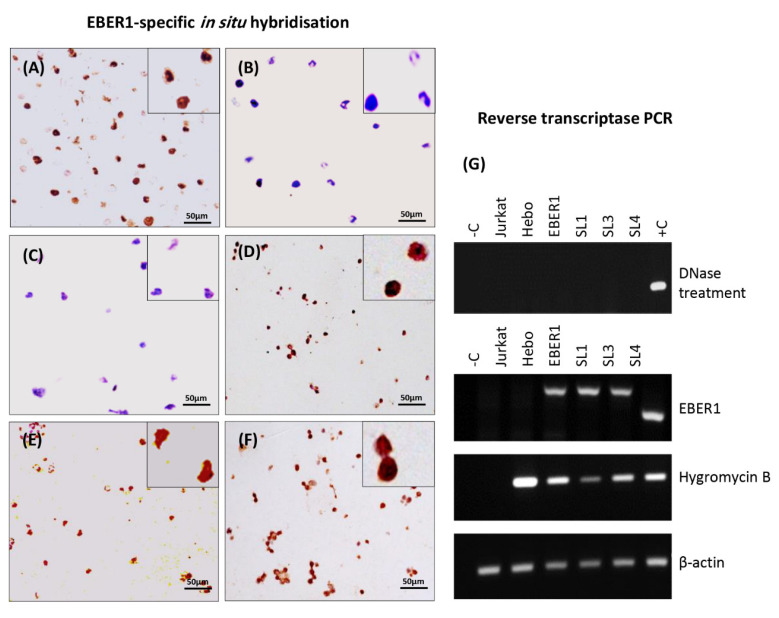
**Confirmation of successful transfection**. EBER1 expression in transfected cells was confirmed by EBER1-specific in situ hybridisation. (**A**) Strong nuclear signal (brown) was observed in EBV-infected cell line, B95.8, used as a positive control. (**B**) Untransfected cells or (**C**) cells transfected with pHebo (plasmid without the EBER1 insert) were clearly negative for EBER1 expression (blue/purple). Cell transfected with (**D**) wildtype EBER1 or (**E**) with ∆SL1 or (**F**) ∆SL3 were also clearly positive for EBER1. (**G**) RT-PCR was used to independently confirm the uptake of the plasmid. Total cellular RNA was isolated using the TRIzol method, and DNase treated to ensure no DNA contamination. A total of 5 µg of the DNase-treated sample was reverse transcribed to cDNA, and 50 ng was used for the RT-PCR to amplify EBER1 gene, Hygromycin B gene, and β–actin—housekeeping and loading control.

Using sequencing, we confirmed the deletion of the desired stem-loop sequences from EBER1 wildtype, creating the three mutants (Figure S1). The wildtype EBER1 and the deletion mutants had similar secondary structures. This structure accurately maps to the chemically probed established structure of EBER1 [30]. EBER1-ISH was used to check EBER1 expression in transfected cells. Strong nuclear signal was observed in EBV-infected cell line, B95.8, used as a positive control (Figure 2A). Cells transfected with pHebo (plasmid without the EBER1 insert) or the untransfected cells were consistently negative for EBER1 expression (Figure 2B,C). All EBER1 (wildtype and mutants) cell lines grew in hygromycin B-supplemented media and were positive for the expression of EBER1 (Figure 2D–F). RT-PCR analysis further confirmed the expression of EBER1 in both wildtype and deletion mutants (Figure 2G). qRT-PCR analysis indicated that there were 0.161, 0.062, 0.054, and 0.104 EBER1 copies/µL of DNA in the wildtype, ∆SL1, ∆SL3, and ∆SL4 transfectants, respectively. We normalised the viable cell count with these copy numbers to eliminate (if any) interference of EBER1 copies with its structural impact on its induced proliferation.

### 3.2. The Structure of EBER1 Is Important in Inducing Proliferation

**Figure 3:**
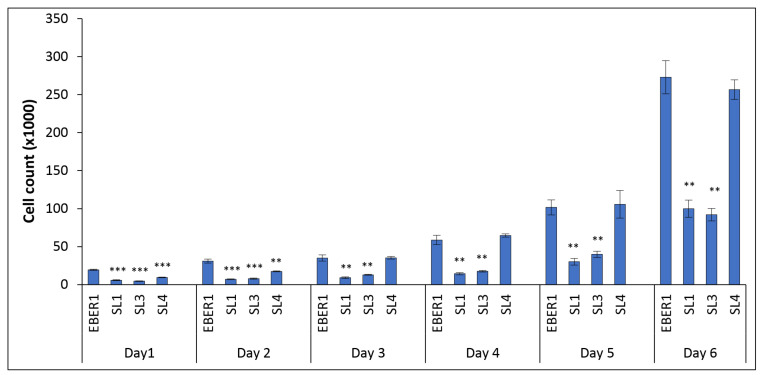
**Impact of EBER1 structure on cell proliferation**. Cell growth was monitored daily with trypan blue exclusion dye. The absolute viable cell count was normalised to the respective copy number of each construct. The result showed that ∆SL1 and ∆SL3 transfectants have significantly less cell growth compared to the wildtype EBER1. Each experiment was performed in duplicate and repeated four times. Data are expressed as mean (±SEM) of the four independent experiments, ** = *p* ≤ 0.01, and *** = *p* ≤ 0.001.

All cells grew normally, as expected. However, there was a significant increase in the rate of proliferation of wildtype EBER1 transfected cells compared to the pHebo negative control (Figure S2). Since the number of plasmids taken up during transfection can vary and thus impact cell proliferation, we normalised cell proliferation with their respective plasmid copy numbers. After normalisation, ∆SL1 and ∆SL3 were observed to have significantly reduced growth rates compared to the wildtype (Figure 3). ∆SL4, on the other hand, was not significantly different from the wildtype (Figure 3). Statistically, the overall structure of EBER1 had a significant role in cell proliferation (*p* = 0.045).

### 3.3. EBER1-Induced Cell Proliferation Is Not Affected by Cell Death or DNA Repair

**Figure 4:**
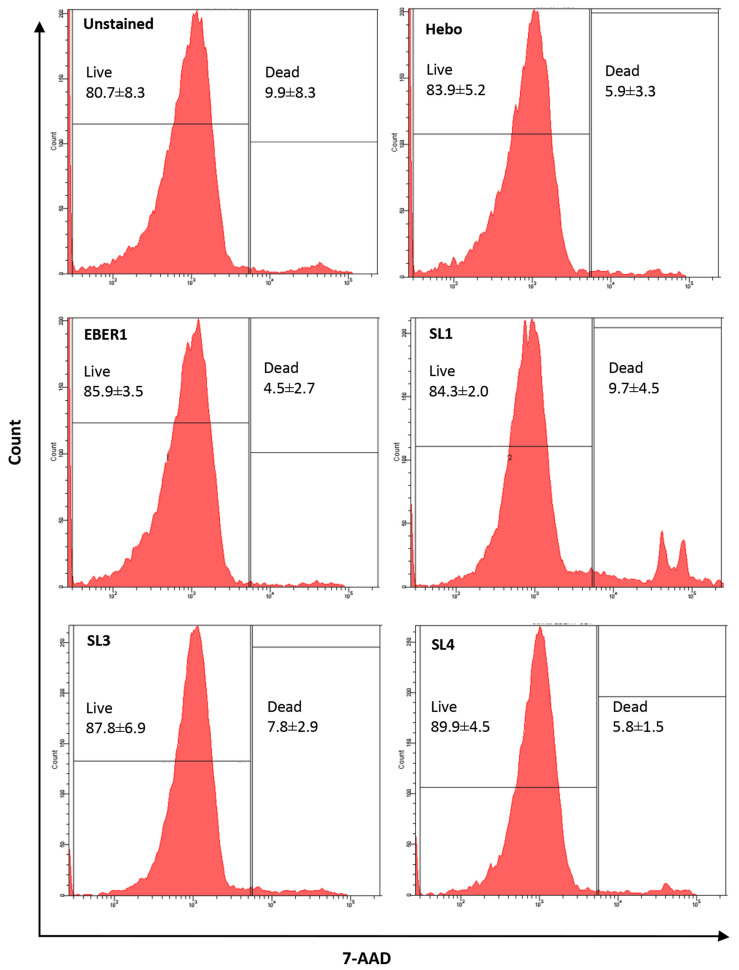
**Cell viability of transfected cells**. Percentage viability was determined by 7AAD dye. There was no significant difference in the rate of cell death between cell lines. Data are presented as mean (±SD) of the four independent experiments.

To ensure that the difference in cell proliferation between wildtype EBER1 cells and the mutants was not due to differences in cell deaths, we stained the cells with 7AAD viability dye and analysed them using flow cytometer [31]. The percentage of cell viability was found to be comparable in all cells, more than 80%, at day 6 of culture (Figure 4). 

**Figure 5:**
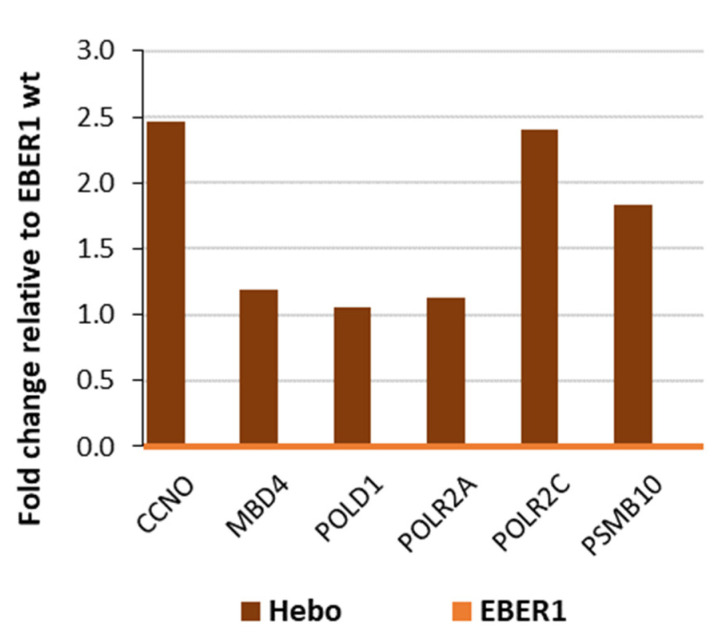
**Analysis of genes involved in DNA repair**. Total cellular RNA was isolated using TRIzol method, and a total of 5 µg was reverse transcribed to cDNA. A total of 92 genes associated with DNA repair were quantified using a qPCR-based microarray plate. There was no gene observed to be up to 1-fold decreased. However, pHebo cells showed upregulation of some genes. EBER1 wildtype is set at 0.0 as reference.

Repair of damaged DNA is another factor that could slow down cell division. Therefore, we quantified the expression of genes responsible for DNA damage repair using a qPCR-based microarray plate. A total of 92 genes associated with DNA repair were analysed, and at least 1-fold change (relative to wildtype EBER1) was considered significant. There was no gene observed to be up to 1-fold decreased (Figure S3). However, pHebo cells showed significant overexpression of a few genes, most notably CCNO, POLR2C, and PSMB10, compared to the wildtype EBER1 cells (Figure 5). Since most of these genes are not exclusive to DNA repair and are also involved in proliferation, it is difficult to conclude if this upregulation is because of DNA repair or proliferation. To clarify this, we studied the mechanisms of cell cycle proliferation and regulation.

### 3.4. EBER1 Transfected Cells Are More in G2/M Phase

**Figure 6:**
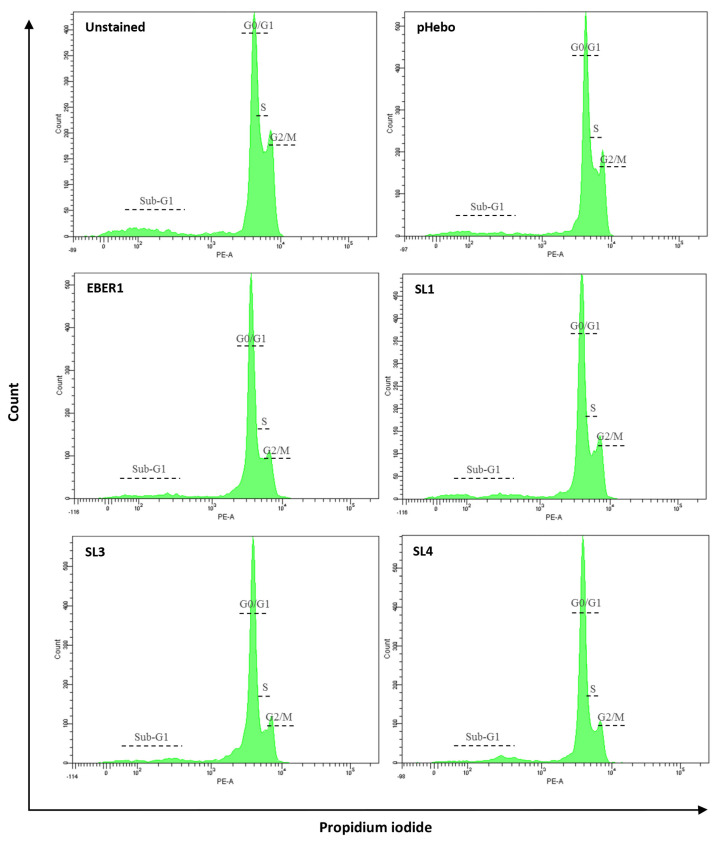
**Percentage distribution of cells across the cell cycle**. Flow cytometric analysis of cells taken on day 6 of culture and stained with PI. Ten thousand events were recorded per sample. Experiments were performed in duplicate and repeated four times.

The distribution of DNA contents in the cell cycle phases was determined by flow cytometry following PI staining. We observed that nearly all the cells were in the cell cycle, and very few were in the sub-G1 phase (Figure 6). From the distributed DNA content, pHebo, ∆SL3, and ∆SL4 had more DNA in the preparatory G0/G1 phase (Table 1). While we did not see much difference in the pattern of cell cycle progression in the S-phase, the wildtype EBER1 cells had a higher G2/M phase population compared to the pHebo (Table 1). 

### 3.5. Genes Involved in Cell Cycle Progression Are Upregulated in Wildtype EBER1

**Figure 7:**
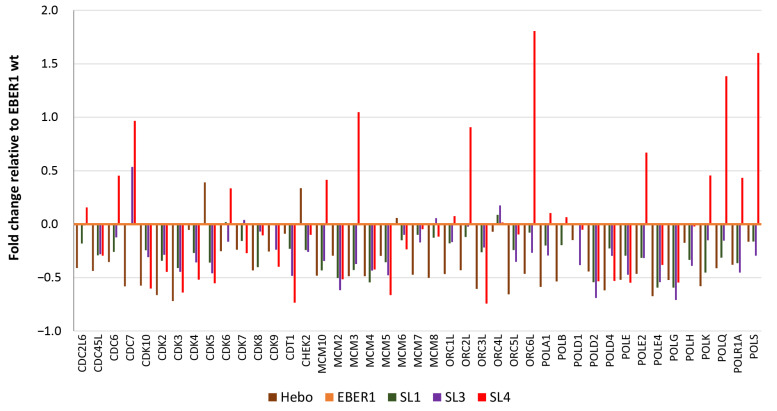
**Analysis of genes involved in cell cycle and proliferation**. Total cellular RNA was isolated using TRIzol method, and a total of 5 µg was reverse transcribed to cDNA. A total of 43 genes associated with human cell cycle and chromosomal replication were quantified using qPCR-based microarray plate. There was no gene observed to be up to 1-fold decreased. ∆SL4 cells showed upregulation in some genes. EBER1 wildtype is set at 0.0 as reference. Experiments were performed in duplicate. Data are presented as mean of two experiments.

Using a qPCR-based microarray plate, we screened 43 genes (in duplicates) associated with cell cycle and proliferation. At least 1-fold difference relative to the wildtype EBER1 was considered significant for analysis. Most of the genes were downregulated in pHebo, ∆SL1, and ∆SL3 cells compared to wildtype EBER1 (Figure 7). ∆SL4 mutant, on the other hand, had up to 1-fold increased expression of some genes, namely, MCM3, ORC6L, POLQ, and POLS (Figure 7). These genes are involved in identifying the origin of replication or DNA replication. Thus, they are expressed in G1 phase, G1/S phase transition, or during the S-phase. Therefore, we studied the cell cycle regulations in these phases.

### 3.6. EBER1 Upregulates Genes Involved in Cell Cycle Progression and Proliferation 

**Figure 8:**
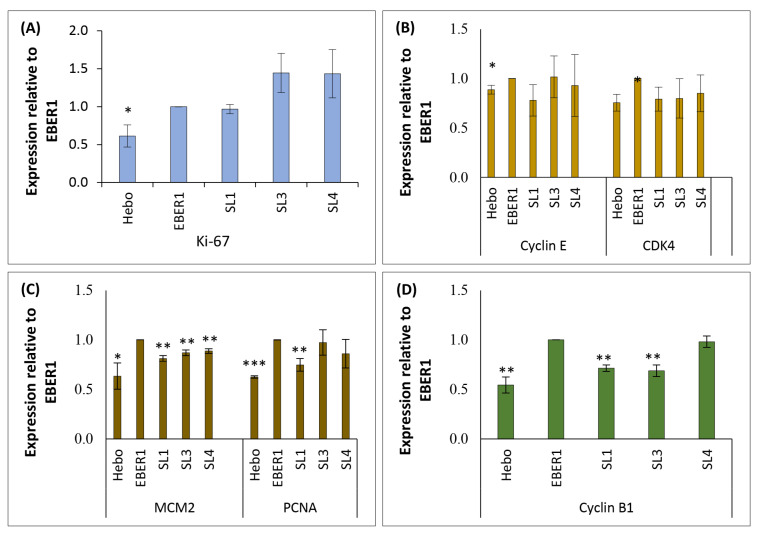
**Quantitative expression of genes associated with cell cycle progression**. Total cellular RNA was isolated using TRIzol method, and a total of 5 µg was reverse transcribed to cDNA. A total of 100 ng was amplified in a qRT-PCR reaction for the quantification of cell cycle genes. (**A**) Pan cell cycle marker. (**B**) Genes expressed in G0/G1 phase. (**C**) Genes expressed in S phase. (**D**) Genes expressed in G2/M phase. Each experiment was performed in duplicate and repeated three times. Data were expressed as mean (±SEM) of the three independent experiments. * = *p* ≤ 0.05, ** = *p* ≤ 0.01, and *** = *p* ≤ 0.001.

Following the microarray screening for cell cycle and proliferation, we used qRT-PCR to examine the expression of specific genes involved in this process, namely Ki-67, cyclin E, CDK4, MCM2, PCNA, and cyclin B1. The data indicated that these genes were expressed at a significantly higher level in wildtype EBER1 compared to the pHebo (Figure 8). The mutants, especially ∆SL1, showed decreased expression of G0/G1 phase markers, but this was not statistically significant (Figure 8B). However, there was significantly reduced expression of S-phase markers in ΔSL1 mutant (Figure 8C). It was also observed that deleting SL1 and SL3 but not SL4 of EBER1 resulted in a significantly decreased expression of G2/M phase markers (Figure 8D). 

### 3.7. Mechanism of EBER1-Induced Cell Proliferation Appears to Involve CDT1 Upregulation

**Figure 9:**
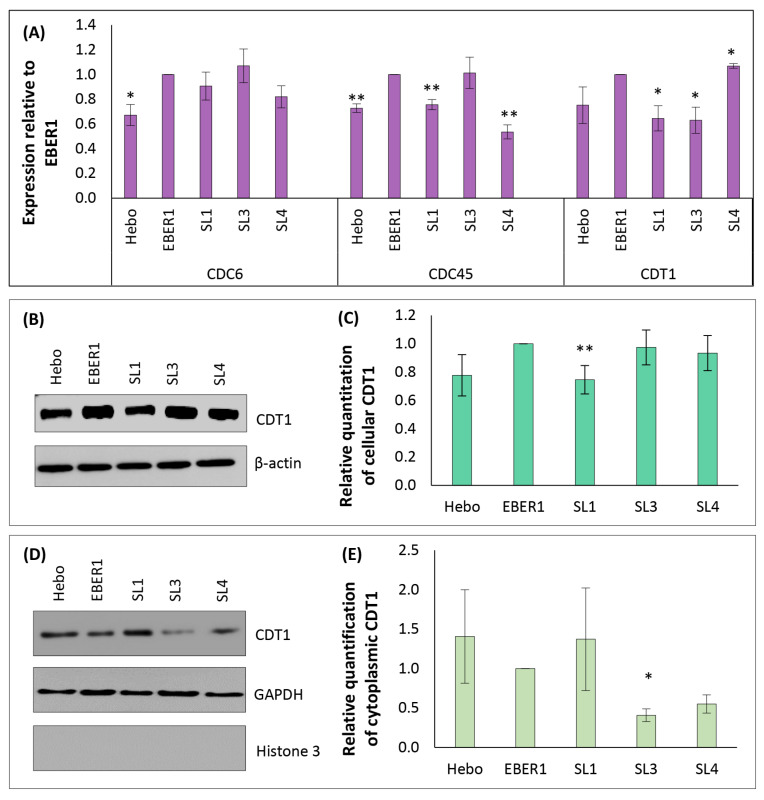
**Expression of markers involved in cell cycle regulation in EBER1 transfectants**. (**A**) qRT-PCR expression of regulatory genes necessary for firing an origin of replication (from the day 6 cells count). Expression of CDT1 protein from total cellular proteins obtained from the day 6 cell count was separated on an SDS-PAGE. (**B**) Western blot image from total cellular protein. (**C**) ImageJ quantification of total cellular CDT1 Western blot bands. (**D**) Western blot image of cytoplasmic CDT1. (**E**) Relative quantification of cytoplasmic CDT1 by qRT-PCR. Each experiment was performed in duplicate and repeated three times. Data were expressed as mean (±SEM) of the three independent experiments relative to EBER1 wildtype. * = *p* ≤ 0.05, ** = *p* ≤ 0.01.

Subsequently, we studied the mechanisms of cell cycle regulation that are found mainly in the G0/G1 or transition to S-phase. Specifically, we looked into the expression of genes involved in the assembly of prereplication complex. We found significantly increased expression of CDC6, CDC45, and CDT1 in wildtype EBER1 compared to the pHebo negative control (Figure 9A). We then focused on CDT1 protein since it plays a central role in firing and regulating the origins of replication [32]. The expression of CDT1 protein in pHebo and ∆SL1 was found to be less compared to the wildtype EBER1 cells (Figure 9B,C). Furthermore, the two (pHebo and ∆SL1) had higher cytoplasmic expression of the protein compared to the wildtype EBER1 cells (Figure 9D,E), but this was not statistically significant. To confirm that SL1 was important in regulating CDT1 expression and localisation, and in EBER1-induced cell proliferation, we tested its impact on another cell line, HEK293T (an epithelial origin). We found that ∆SL1 mutant indeed had significantly lower expression of CDT1 at the whole cell level and higher expression in the cytoplasm. Furthermore, this mutant, ∆SL1, had lower cell growth compared to the wildtype EBER1 (Figure S4).

## 4. Discussion

Epstein–Barr virus is associated with many types of human cancers of lymphoid and epithelial origins. In 2017, over 265,000 incident cases and 164,000 deaths were attributed to EBV-associated malignancies, and this burden appears to be increasing [33]. The virus preferentially and efficiently infects and establishes latency in the lymphocytes [34]. Thus, lymphocytes can support both latent and lytic EBV infection. Epithelial cells, on the other hand, are primarily involved in the lytic cycle and aid amplification and transmission of the virus [35,36,37]. Most EBV-associated cancers are linked to latent infection [2,36]. Depending on the pattern of latent gene expression, EBV latency is divided into four programs (latency 0, I, II, and III) [2]. EBV-encoded RNAs are highly expressed in all four EBV latent programs. 

EBERs are two small, noncoding, and structurally conserved transcripts. One of them, EBER1, has 167 nucleotides corresponding to positions 6629 to 6796 on the EBV genome. It folds into four stem-loop structures by intramolecular base pairing [30]. Noncoding RNAs are known for regulating cellular processes. For example, they serve as epigenetic regulators that modulate cellular chromatin by aiding and ensuring specific binding of a target molecule [38,39]. For instance, EBERs recruit PAX5 transcription factor to EBV DNA [7,39,40]. In addition, EBERs alter cellular and viral gene expressions [7,11,41]. Similarly, EBERs are involved in conferring resistance to innate immune antiviral processes [10,41,42]. EBERs exist as ribonucleoproteins, interacting with several cellular proteins. It has been hypothesised that the interaction of these RNAs with cellular proteins drives many of their functions, including facilitating cell transformation [11,14]. 

Several studies reported that EBERs are associated with enhanced cell proliferation, both in vitro and in vivo [14,43,44]. Although EBER1 is often studied in conjunction with EBER2, the growth-promoting properties are linked to EBER1 [14]. In the present study, we investigated the potential mechanism of EBER1-induced cell growth in a lymphoid cell line and the role of EBER1 conserved structure in the proliferation process. We found that the structure of EBER1 is important for its induced cell proliferation in Jurkat cell line. Deletion of SL1 and SL3 of EBER1 significantly decreased cell growth compared to the wildtype EBER1. By contrast, deleting SL4 had no effect.

EBER1 binds to several proteins, such as PKR, RPL22, and telomerase RNA [44]. These proteins contribute to cell proliferation. SL1, SL3, and SL4 investigated in this study have been shown to bind RPL22 [45,46]. Furthermore, the interaction between EBER1 and RPL22 was shown to be essential for proliferation and tumorigenicity of EBV-infected Burkitt’s lymphoma cells [14]. It was also observed that increased cell growth was abolished in cells expressing EBER1 with mutations in RPL22 binding sites, even in the presence of wildtype EBER2 [14]. Similarly, in the present study, we found that cell proliferation was decreased in cells transfected with SL1- and SL3-deleted EBER1.

Other potential mechanisms of EBER1-induced cell proliferation include the activation of PI3K/Akt and IL-6/STAT3 pathways [9,47]. IL-6/STAT3 pathways downregulate cell cycle inhibitory genes, p21 and p27, thus indirectly promoting the activity of CDK2 and CDK4, leading to G1/S transition [47,48]. Our observations from this study are in line with these findings. We observed a significantly increased expression of the G1/S transition phase markers, such as CDK4, in the wildtype EBER1 compared to the negative control. This increase suggests an early transition which appeared to subsequently lead to significantly increased expression in all the cell cycle stages. A normal healthy cell ensures only one round of DNA replication per cycle. CDKs interact with several cyclins at various gap stages within the cell cycle. For a cell to commit to replication, CDK4/6 associates with D-type cyclins to drive the G1/S-phase transition where DNA is replicated [49]. 

There are a set of regulations that govern this critical cell physiology. These regulations include licensing the origin of replication only once per cell cycle. This is achieved by cytoplasmic translocation, inhibiting or autodegrading some essential components required for firing the origin of replication, such as the CDT1 [32,49]. Multiple firing of the origin of replication can lead to many defects, including chromosome rearrangements, partially re-replicated DNA, and accumulation of short dsDNA and ssDNA [49,50]. These defects, over time, may lead to genomic instability, an important hallmark of cancer [49,50]. In this study, we showed that EBER1 increased the expression of CDT1 protein compared to the negative control cells and thus promoted cell proliferation. In ∆SL1 mutant, CDT1 expression was similar to that in negative control cells and had a comparable growth rate. To confirm that SL1 was important in regulating CDT1 expression, we investigated the same in HEK293T transfected cells. We found that ∆SL1 mutant indeed had significantly less CDT1 expression, and less cell growth compared to the wildtype EBER1. Furthermore, following the G1/S transition, we observed a significant increase in the expression of S-phase markers in the wildtype EBER1 cells. This supports our earlier report that wildtype EBER1 prolongs S-phase in the lymphoid cells [13]. Abnormal gene expression is an initial trigger of cell cycle disorders, which eventually could lead to malignancies [47]. Although there are several mechanisms for a cell to rectify and repair damaged DNA, these mechanisms may also be deleterious to genome integrity [50]. Moreover, these mechanisms, including DNA repair and the rate of cell death, were not found to impact cell proliferation.

Although the findings of this study indicate that SL1 plays a role in cell proliferation, further work is needed to confirm and elucidate the potential mechanism involved. This study was limited to investigating the impact of the different stem-loops of EBER1 on cell proliferation in T cells. Considering that EBV is a B-cell tropic virus, it is important to verify these findings in a B-cell line. Finally, the impact of EBER1 on transfected cells could be very different from EBV-infected cells in vivo, where a number of viral and cellular factors could be interacting.

## 5. Conclusions

The mechanism of EBER1-induced cell proliferation could be by infringing cell cycle regulations found in the G1-phase. This in turn facilitates G1/S phase transition and cell division. Deleting SL1 abolished EBER1-induced proliferation and, thus, seemed to restore normal cell growth. The structure of EBER1, especially SL1, appears to be important in its induced cell proliferation.

## Figures and Tables

**Table 1:** **Percentage distribution of DNA contents across the cell cycle**. The percentage distribution of DNA contents in the cell cycle phases (G0/G1, S, and G2/M) was analysed using FlowJo. Experiments were performed in duplicate and repeated four times. The data are presented as mean of the four independent experiments. The table is colour formatted from red (lower percentage) through green (higher percentage).

	% G1	% S	% G2/M
Hebo	43.4	26.4	34.0
EBER1	30.9	27.8	43.0
SL1	36.5	25.3	41.5
SL3	45.8	26.5	35.9
SL4	45.6	30.9	30.4

## Data Availability

Not applicable.

## Supplementary Materials

The following supporting information can be downloaded at: https://www.mdpi.com/article/10.3390/v14112538/s1, Figure S1: Sequence alignment of EBER1 wildtype and mutants; Figure S2: EBER1 increases the rate cell growth compared to control cells; Figure S3: Analysis of genes involved in DNA repair; Figure S4: Impact of SL1 deletion on proliferation in HEK293T cells; Table S1: List of primers used in this study.
