# Supplementary material for: The Impact of Deleting Stem-Loop 1 of Epstein–Barr Virus-Encoded RNA 1 on Cell Proliferation
Source: Viruses. 2022 Nov 16;14(11):2538. doi: 10.3390/v14112538 (PMC9696203; doi:10.3390/v14112538)
Supplement: Supplementary file 1 [file viruses-14-02538-s001.zip › viruses-1991057-supplementary.pdf]

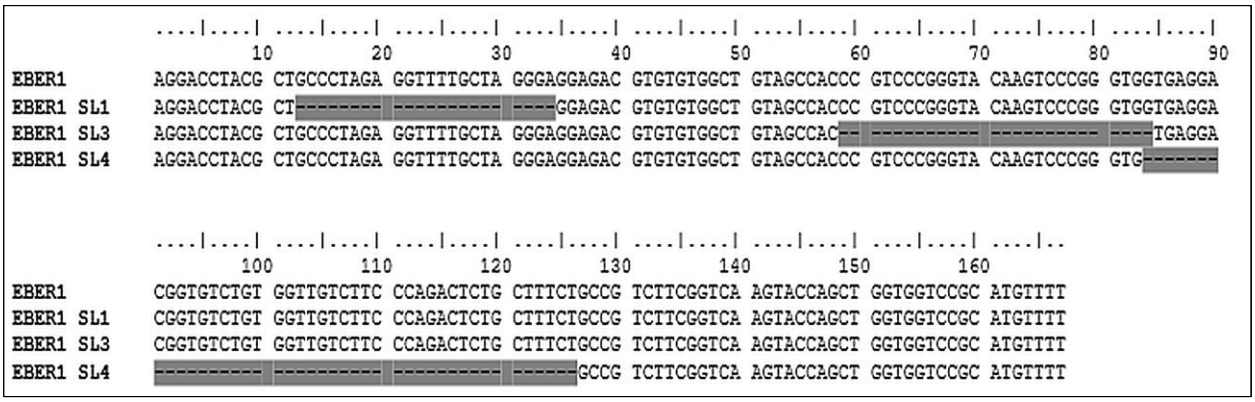

Figure S1. Sequence alignment of EBER1 wildtype and mutants. Sequence deletions in EBER1 were confirmed by Sanger sequencing and aligned in BioEdit™ software. The sequence alignment revealed the deletion of sequences corresponding to nucleotide positions 13-34 (22 nt), 59-84 (26 nt), and 84-126 (43 nt). Thus, forming SL1, SL3 and SL4 mutants. The grey-shaded portion in each mutant indicates the deleted sequence positions.

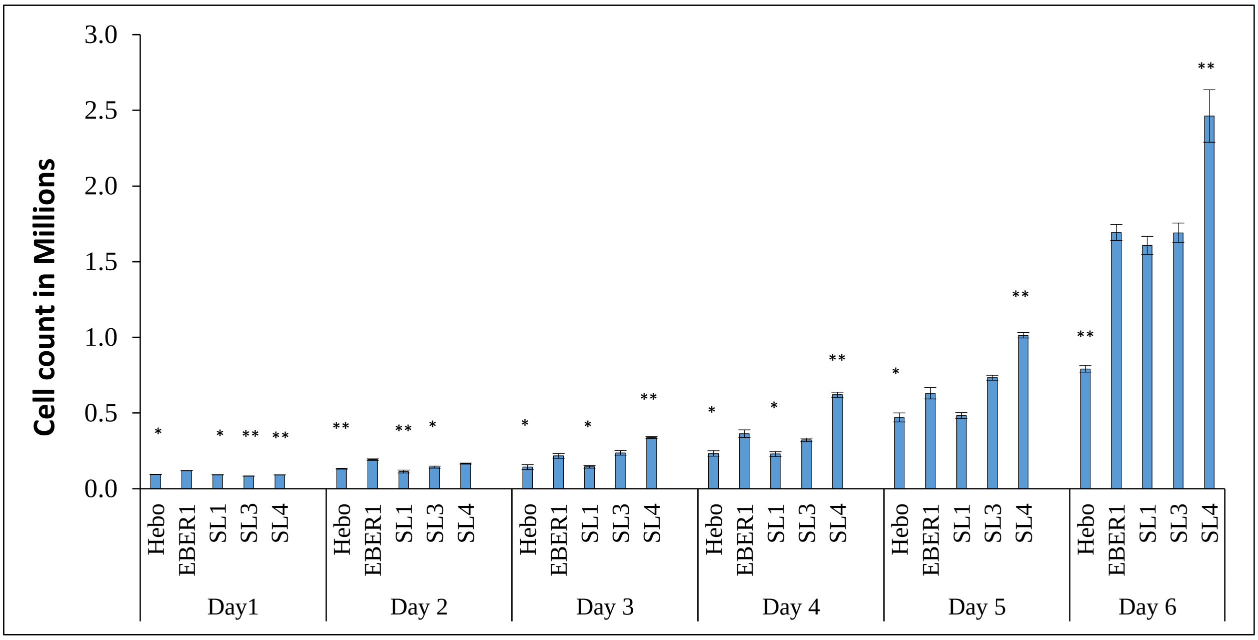

Figure S2: EBER1 increases the rate cell growth compared to control cells. Cell growth was monitored daily with trypan blue exclusion dye. The result showed that EBER1 increases cell growth compared to the pHebo control cells. Each experiment was done in duplicate and repeated four times. Data are expressed as mean (±SEM) of the four independent experiments. *= $p \leq 0.05$, **= $p \leq 0.01$.

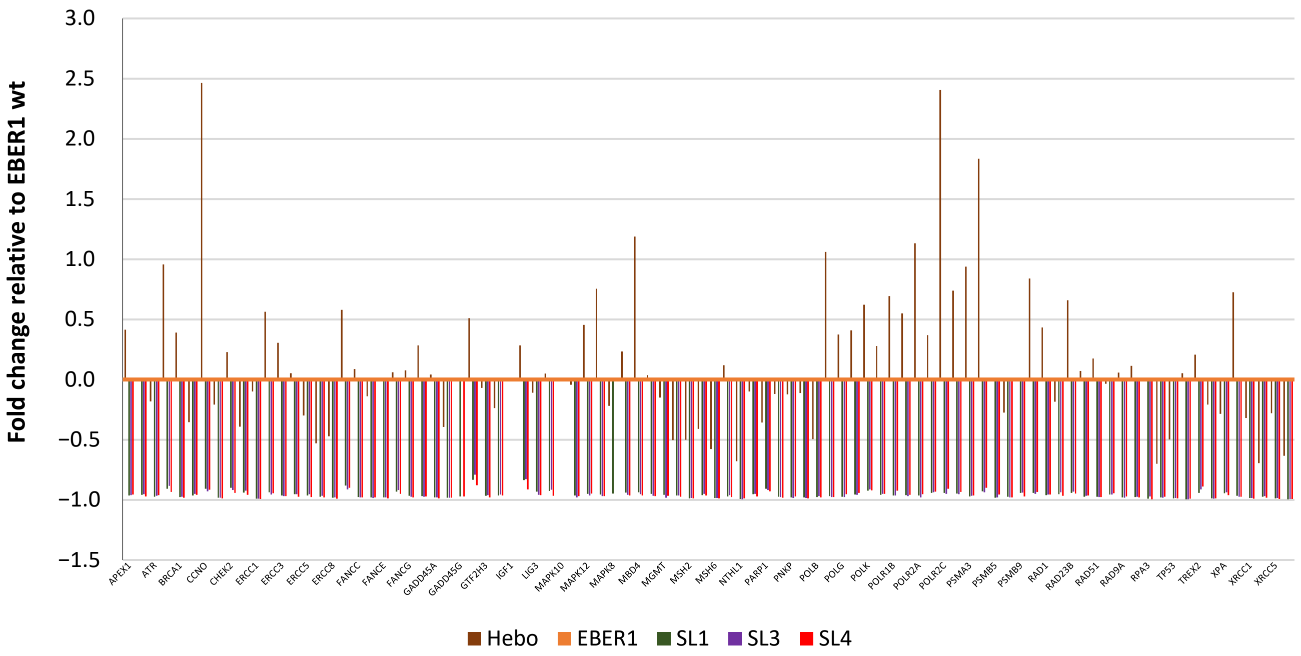

Figure S3. Analysis of genes involved in DNA repair. Total cellular RNA was isolated using TRIzol method, and a total of 5 μg was reverse transcribed to cDNA. A total of 92 genes associated with human cell cycle and chromosomal replication were quantified using qPCR-based microarray plate. No gene observed to be up to 1-fold decreased. pHebo cells showed upregulation of CCNO, MBD4, POLD1, POLR2A, POLR2C and PSMB10 genes. EBER1 wildtype was set at 0.0 as reference.

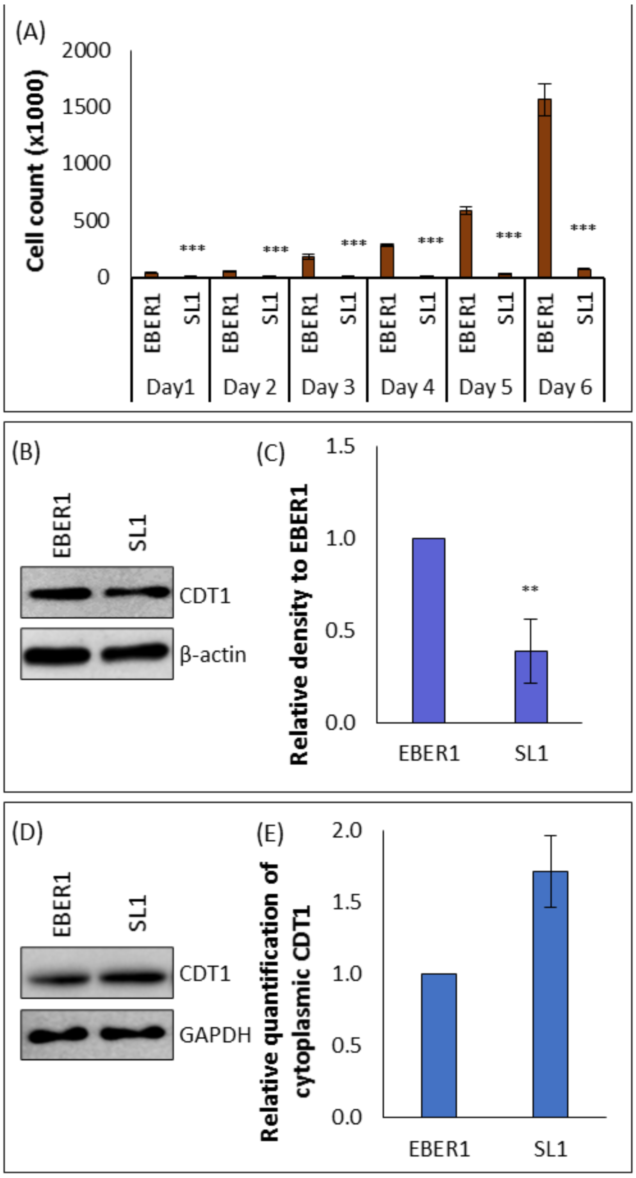

Figure S4. Impact of SL1 deletion on proliferation in HEK293T cell line. (A) Copy number-normalised viable cell count in HEK293T cell line. There was significantly decreased rate of cell growth in SL1 cell line compared to the wildtype EBER1 cell line and all days. Proteins obtained from day 6 cell count were separated on an SDS-PAGE. Total cellular expression of CDT1, (B) Western blot image and (C) ImageJ quantification. Cytoplasmic expression of CDT1, (D) Western blot image and (E) qPCR quantification of cytoplasmic CDT1 relative to EBER1 wildtype. Experiments were performed in duplicate and repeated three times. Data are expressed as mean (±SEM) of the three independent experiments. *= $p \leq 0.05$, **= $p \leq 0.01$, and ***= $p \leq 0.001$.
